# Mobility Infrastructures and Health: Scoping Review of studies in Europe

**DOI:** 10.3389/phrs.2024.1606862

**Published:** 2024-05-22

**Authors:** Sarah Michel, Nicola Banwell, Nicolas Senn

**Affiliations:** ^1^ Department of Family Medicine, Center for Primary Care and Public Health (Unisanté), University of Lausanne, Lausanne, Switzerland; ^2^ Interdisciplinary Centre for Research in Ethics (CIRE), University of Lausanne, Lausanne, Switzerland

**Keywords:** mobility, active transportation, health, behaviour change, co-benefits, active mobility, walking, cycling

## Abstract

**Objectives:**

Movement-friendly environments with infrastructure favouring active mobility are important for promoting physical activity. This scoping literature review aims at identifying the current evidence for links between mobility infrastructures and (a) behaviour regarding active mobility, (b) health outcomes and (c) co-benefits.

**Method:**

This review was conducted in accordance with the PRISMA scoping review guidelines using PubMed and EMBASE databases. Studies included in this review were conducted in Europe, and published between 2000 and March 2023.

**Results:**

146 scientific articles and grey literature reports were identified. Connectivity of sidewalks, walkability, and accessibility of shops, services and work are associated with walking. Cycling is positively associated with cycle-paths, separation of cycling from traffic and proximity to greenspaces, and negatively associated with traffic danger. Increased active transportation has a protective effect on cardiovascular and respiratory health, obesity, fitness, and quality of life. Co-benefits result from the reduction of individual motorized transportation including reduced environmental pollution and projected healthcare expenditure.

**Conclusion:**

Mobility infrastructure combined with social and educational incentives are effective in promoting active travel and reducing future healthcare expenses. A shift to active transportation would increase both individual and community health and decrease greenhouse gas emissions.

## Introduction

Movement-friendly environments, in particular infrastructure that favours active mobility, are important leverage points for promoting physical activity and subsequent health outcomes [1, 2]. The World Health Organization (WHO) guidelines on physical activity and sedentary behaviour recommend daily doses of both moderate to vigorous physical activity (MVPA), and vigorous activity for all age categories (e.g., adolescents should do at least 60 min/day of MVPA) to support health benefits and protect against health risks [3]. The guidelines report that the health benefits of such activities include, among others, improved physical fitness and cardiometabolic health for children and adolescents, and reduced cardiovascular disease mortality for adults and older adults. However, the 2019 Switzerland physical activity fact sheet reported that about 60% of adolescents, and at least 25% of adults older than 35 years old were not meeting sufficient activity levels [4]. Replacing daily activities such as transportation by their more active counterpart is an interesting strategy to promote physical activity so that more people attain the recommended levels, because this potentially allows common obstacles such as time constraints to be overcome. Meanwhile, the transport sector accounts for 30% of the greenhouse gas (GHG) emissions of Switzerland, with the transport of people representing about 73% in 2021 [5]. A modal shift towards active mobility (walking and cycling) would allow for a win-win situation in terms of human health and environmental benefits. Such win-win interventions, referred to within this review as co-benefits, have simultaneous positive impacts for human health and the environment [6]. Urban planners and stakeholders involved in infrastructure management have important roles to ensure the future development of physical environments that promote such co-benefits [2]. Understanding the effectiveness of active mobility infrastructure for promoting health and co-benefits, as well as the identification of specific interventions is important for health promotion and urban policy to promote good physical and mental health while also protecting the environment.

The objective of this scoping review is to identifying the current evidence for links between mobility infrastructures and (a) behaviour regarding active mobility, (b) health outcomes and (c) co-benefits.

## Methods

The scoping review focused on mobility infrastructure and three specific outcomes of interest, including a) impacts on behavioural change, b) impact on physical and mental health, and c) co-benefits. Mobility infrastructure here refers to built infrastructures that support and facilitate physical movement, exercise and activity. It includes features such as accessible paths for cycling and walking, and areas specifically designed for physical activity such as playgrounds. By behaviour change we refer to the uptake of a new practice, e.g., cycling and walking instead of using riding by car, as well as the reinforcement of an already adopted practice e.g., more cycling, more walking. We included the use of electrically-assisted bikes, because they still require a certain level of physical efforts. Programs promoting active mobility (e.g., promotion of cycling at school) were considered as complementary strategies to mobility infrastructure interventions which enable behaviour change. Co-benefits refer to interventions that are simultaneously beneficial for maintaining, restoring or improving both human health and the environment. Within the context of this literature screening, a specific focus is placed on co-benefits for climate change and health system expenses.

Alongside this review, a second scoping review was undertaken centered on greenspaces (refer to Banwell et al 2024, titled “Greenspaces and Health: Scoping Review of studies in Europe”). Both of these reviews have been conducted in parallel in accordance with the PRISMA guidelines for reporting scoping reviews [7]. The full search strategies for both reviews were developed separately in collaboration with librarians specialized in health literature searches of Unisanté (University of Lausanne). Concerning the present review, combinations of key search terms such as “built environment,” “city planning,” “environment design,” “urbanization,” “active commuting,” “transportation,” “bicycling,” “pedestrians,” “health behaviour change,” “healthy behaviour,” “physical activity,” “exercise,” among others were used to identify relevant literature (full list available in Supplementary Appendix S1). Databases which were used for the search include PubMed and EMBASE. All the references were extracted on 2 March 2023. The data extraction, identification and analysis were conducted separately for the two reviews. Relevant articles were then selected and classified according to the established criteria (Supplementary Appendix S2) using Covidence^®^ software, including a geographical focus on Europe, and in particular Switzerland, and publication year from 2000 to 2023 at the date of extraction. No criteria related to age group was applied (See Supplementary Appendix). Furthermore, for outcome *c) co-benefits*, the review drew on a literature review previously conducted by members of the research team [2]. In addition to the scientific literature, grey literature from reputable international organisations (see Supplementary Appendix S2) in relevant domains were also identified and included. The strength of evidence was assessed qualitatively based on author consensus where “strong evidence” was considered to refer to cases where systematic reviews and meta-analyses were available.

## Results

**FIGURE 1:**
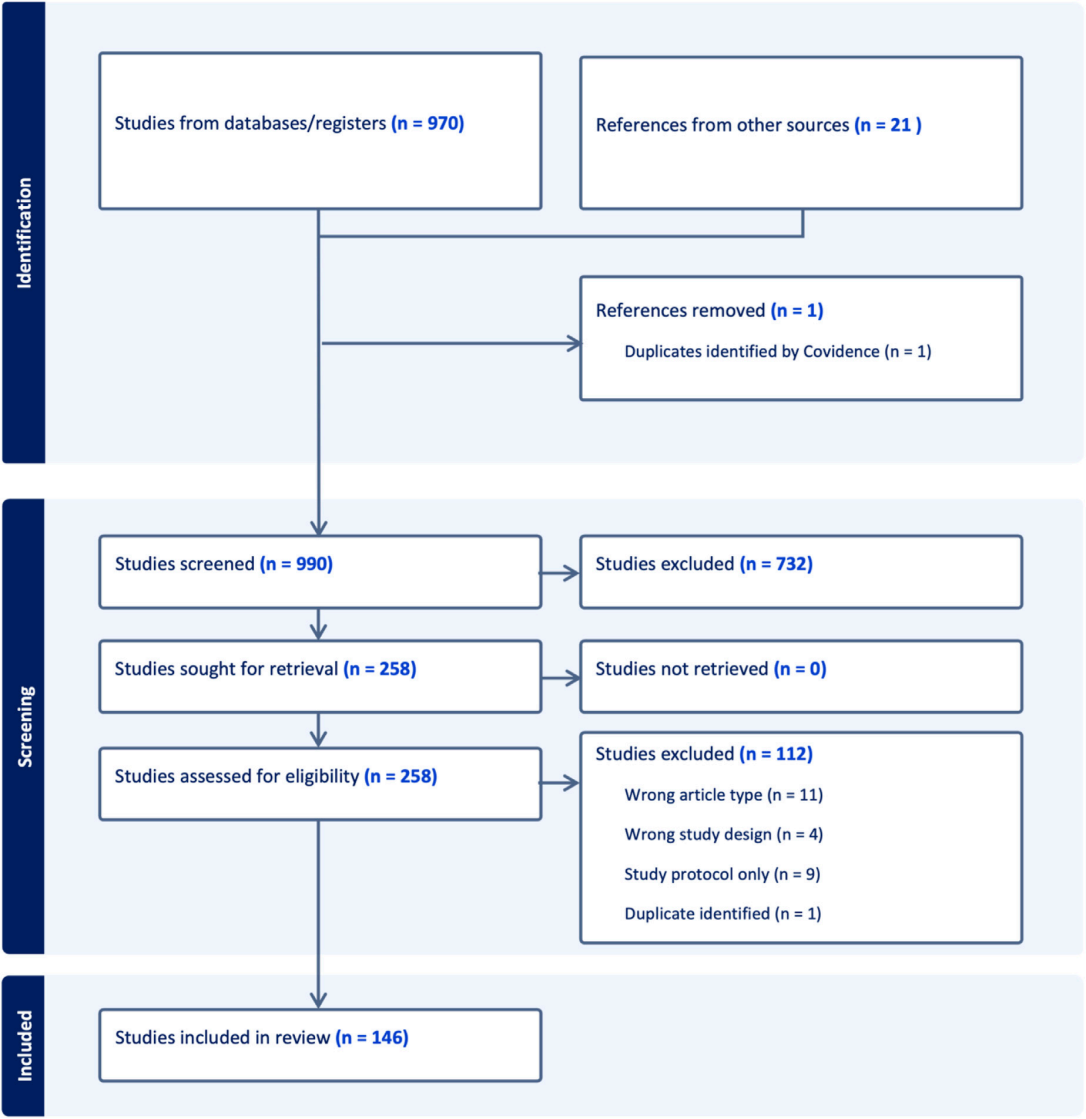
Flow diagram of the scoping review process indicating the number of scientific articles processed (Switzerland, 2024). Initially 970 studies were identified and 21 additional references pertinent for the European context were added, including scientific studies and reports from reputable non-governmental and government organisations. At the end of the abstract and full-text screening 146 studies met the established criteria for inclusion in the scoping review (criteria provided in the Supplementary Material).

The scoping review identified a total of 146 combined scientific articles and grey literature reports. The articles that were included through the search examined the relationship between mobility infrastructure interventions and the three outcomes of interest. Among those, 21 were included through citations and grey literature searches. Figure 1 provides an overview of the number of articles and grey literature included in the review. Table 1 shows the article types included in the review. Table 2 displays the types of mobility interventions (either directly related to the mobility infrastructure, or to a mobility behaviour) that were identified.

**TABLE 1:** Types of articles identified (Switzerland, 2024).

Type of article	No of articles total = 125
*Literature reviews*	*46*
Systematic reviews	22
Scoping reviews	9
Narrative reviews	5
Umbrella reviews	2
Rapid reviews	2
Reviews of miscellaneous types	6
*Cross sectional studies*	43
*Randomized control studies*	18
*Modelling studies*	8
*Cohort studies*	6
*Prospective studies*	3
*Guidelines*	1

**TABLE 2:** Identified mobility interventions (Switzerland, 2024).

Intervention type	Corresponding studies
Built environment	
*Human-made features and physical infrastructure in which people live, work and carry out recreational activities*	
Walking infrastructure	(8–21)
Cycling infrastructure	(22–35)
Walking and cycling infrastructure	(36–46)
Access and proximity	(12, 14, 20, 28, 47–53)
*The distance between the current location and target location as well as the ease with which it can be covered*	
Play-infrastructure	(40, 52, 54–56)
*Includes for example, playgrounds, outdoor areas facilitating children’s play, as well as sports facilities*	
Car-related infrastructure	(43, 44, 57–60)
*Includes for example, car parking, crossroads and busy roads*	
Pollution and noise exposure	(51, 61–79)
*Exposure to particulate matter less than 2.5 µm and to traffic noise exceeding national guidelines*	
Physical activity and active travel	
Travel to school	(47, 80–94)
Commuting to work	(57, 95–112)
Physical activity	(61–64, 113–137)
Confounding factors	
Income and socioeconomic status	(16, 17, 19, 37, 42, 48, 86, 93, 95, 138–140)
Gender	(23, 27, 39, 53, 92)
Ethnicity	(91)

### Behaviour Change

In the identified literature, the link between mobility infrastructure and behaviour change was most often analyzed within cross-sectional studies. A few longitudinal studies reported on the implementation of heavy infrastructural changes such as bike lane construction or reallocation of road space. In addition, the promotion of active travel and educational programs aiming at active travel behaviour were reported. Active travel was thus reported either as a result of specific physical environment or social incentives. The features that were most often associated with active behaviour were built environment interventions, proximity, promotion and education, the socio-economic environment, and a weak car-culture. Briefly mentioned here is also that mobility-related physical activity was reported to increase overall physical activity.

#### Effect of Built Environment Interventions on Active Travel

Walkability, a composite measure used to rate the extent of a pedestrian-friendly environment, was found to be positively associated with active transportation across all age groups within the population [8–11, 113]. In a systematic review and meta-analysis, the difference in the amount of steps per day among adults living in high compared to low walkable areas was reported to be 766 (95% C.I.: 250–1271) representing approximately 8% of recommended daily steps [11]. When examined individually, the characteristics that compose “walkability” have less clear relations with active travel patterns and mixed findings are reported in the literature. For example, it has been found that density, land use mix diversity (residential, commercial, … ), street connectivity, walk/cycle facilities, aesthetics, general safety and traffic safety did not influence active transportation to school (walking and cycling) in Europe [9]. Also, a systematic review focusing on 18–65 year old adults reported that better access to recreational facilities, better aesthetics, and traffic- and crime-related safety were not related to active transportation in Europe, whereas better access to shops, services, or work showed a positive association [10]. This suggests that isolated features of the environment have little effect on the overall behaviour and that cumulation of the features would be the most effective intervention. As an example, a cross-sectional study conducted on older people in Belgium calculated an environmental index based on the following factors: absence of high curbs, presence of different shops and services, presence of benches, presence of crossings, presence of bus stops and street lighting, and safety from crime [12]. For perceived short distances, the more of these features, the higher the probability of older people to walk daily (probability of walking of 0.41; 95% C.I.: 0.39 to 0.43 in presence of all seven environmental factors). For perceived medium distances, combinations of four of these factors showed a significant change in the walking probability (probability of 0.31; 95% C.I.: 0.29–0.33) compared to if none of the features were present (0.22; 95% C.I. 0.16–0.28). For perceived higher distances, the features were no longer correlated with increase in walking. A meta-analysis reported that adults have a better experience when exposed to picturesque sights, detail-rich environment, sufficient legibility and order, trees, natural light and fresh air [13].

Separation of cycling from other traffic, high population density, and proximity of a cycle path or greenspace were reported to be positively associated with cycling behaviour in the overall population [22]. Conversely, perceived and objective traffic danger, and distance to cycle path were negatively related to cycling [22].The following studies illustrate these aspects. In 2021, a 1-km-long cycling route was implemented in the centre of the city of Fribourg (Switzerland) in substitution of the existing parking places. At the 1 year follow-up, a 20% increase in cycling counts was reported on weekdays [23]. In Cambridge, after implementation of a 22-km-long traffic-free walking and cycling route, the people living closest to the new infrastructure were the ones most likely to increase their weekly commuting time, amounting to 1 h and 30 min of additional cycling [36]. In the centre of Lisbon, following a city-wide cycling network expansion, the cycling counts augmented by a factor 3.5 within 1 year [24]. Subsequent deployment of 1,400 bikes in a bike sharing system triggered further growth (by a factor 2.5) of the number of people cycling. However, the study observed that bike sharing stations alone were insufficient to increase cycling levels in locations where no other cycling infrastructures were present.

An example of a broad-scale and long-term intervention are the cycling interventions hosted in 18 towns in England between the years 2005 and 2011 [37]. Interventions were made both in terms of infrastructure changes (including bike parking, and cycling lanes and paths) and educational incentives at the expense of 14£ to 17£ per inhabitant, per year over a period of between 3 and 6 years. The prevalence of cycling to work was reported to increase from 5.8% to 6.8% between 2001 and 2011 and to be significantly higher from the cycling-to-work increase in comparison towns. The difference in absolute percentage point increase (difference-in-differences) in cycling was greatest among the most deprived areas (0.77, 95% C.I.: 0.60–0.94) compared to most affluent areas (0.39, 95% C.I.: 0.19–0.59).

Changes to cycling infrastructure can have a two-fold objective: recruiting new active commuters and/or developing cycling as a permanent habit for transportation, thus minimizing the drop-off rates [38]. Cycling must not only be made possible, but it must be made desirable and attractive [39]. As reported by an Austrian cohort-study, most cyclists favour routes displaying bicycle pathways/lanes, flat roads, and attractive areas instead of the shortest way available [25]. In average, the detour represented 7.6% of the shortest distance, which corresponded to 277 additional meters travelled. In Zürich, implementation of cycling boxes (road marking for left-turning bicycle) increased the perceived safety at the crossing [26]. Objectively measured, the vehicles passing the cyclist indeed respected greater minimal distance after the intervention.

Taking action for improving effective and subjective safety is important also for diversifying the profile of the cycling population. For the same trip, women’s perception of safety tends to be lower than men’s [23]. In London, a study by Aldred et al. compared a road having separated cycle track with two parallel roads without traffic separation [27]. They observed a ten-percentage point difference in the number of female cyclists. Fully separated cycling infrastructure would appear safer to women but also to vulnerable populations such as children and older people.

#### Effect of Proximity on Active Travel

Distance to destination is a major factor influencing the mode of transportation [22, 47, 48, 113]. Living within a 20-min walking distance from school is positively associated with walking to school [113]. In another study surveying adolescents, commuting to school by foot was considered by a majority of adolescent up to a maximal distance of 2.5 km (about 30 min walk) [141]. In the same study, the maximal distance considered for biking to school was 4 km (less than 10 min biking). The greater the distance, the lesser the efficiency of incentives related to environmental factor (e.g., presence of shops, services, benches, and crossings) which are usually positively associated with walking [12]. Following implementation of a bike-sharing system in Spain no behaviour change was observed if the closest station was further than 250 m away from the student’s home [28]. Therefore, distance to the nearest bike-sharing station was also seen as an obstacle.

#### Built Environment Interventions and Physical Activity

Physical activities such as play and sports participation can be modulated via the visual perception of the environment, with aesthetics being positively associated with physical activity [14]. School interventions such as colourful playfields or sports-adapted playgrounds, and access to game equipment were also associated with children’s engagement in MVPA [40]. In the meantime, greening of the school ground was subjectively reported to increase light physical activity (LPA). Differences in types of physical activities depending on the environment were further highlighted by a cross-sectional study in the UK. When children were in buildings or in environments dominated by road and pavements, they engaged significantly more in LPA compared to MVPA [54]. Contrastingly, the activity profile in parks and gardens was seen to be dominated by vigorous physical activity. So-called Play Street interventions (urban interventions consisting in reducing traffic of certain roads to provide safe spaces for children to play near home) have also been evaluated over summer vacations for their effectiveness in increasing children MVPA in Belgium [55]. Compared to children living in control neighbourhoods were Play Streets were not implemented, the children with access to Play Streets displayed one additional hour per week of MVPA and 3 hours and a half less sedentary time during the playtime week compared to the week before intervention. Regarding student’s behaviour, a systematic review also reports that a low compactness index and number of sports facilities were both correlated with increased sport-participation [138]. Simple interventions which do not involve infrastructure changes, such as encouraging the use of stairs while traveling or shopping, has been shown to have little impact on adult’s health-enhancing physical activities in the past [15].

Importantly, the built environment is not the only factor conditioning engagement in physical activities and age-specific behaviours. While walkability can explain the propensity of adults to engage in out-of-home activities, the same metric is not valid for the youth and elderly, a Dutch modelling study reports [16]. The authors suspect that the proximity of parks could be better indictors of those activities.

#### Promotion of Education Interventions in Active Travel

“Safe route to school” projects, which are developments or improvements of cycle and foothpaths, were reported to be positively associated with cycling [22]. One study indeed reported a total of 10% more children biking to school when their home to school route had been equipped to ensure their safety, compared to those children without a safe route. However, as previously mentioned, proximity should also be addressed, notably for students living far from school [80]. Mitigated success of a recent randomized control trial in a Spanish school illustrated that promotional and educational measures alone were not sufficient for achieving behaviour change [29]. While post-intervention evaluation revealed better cycling knowledge, it indicated no change in the actual travel behaviour of the children. In addition, students increasingly considered the built environment as a barrier to walking as a means of transportation. Features of the physical environment that represent a barrier to active commuting from childen’s perspectives were the focus of a systematic review. It reported traffic safety as the most statistically significant barrier, followed by distance, presence of highway and absence of crosswalk, road safety, busy street, no direct route, lack of sidewalks and insufficient crossings or visibility [81].

Children’s perception of the built environment, and their travel behaviour is likely influenced by their parent’s travel behaviour. Parents included in a Norwegian randomized control trial, who were previously cycling less than once a week, were given access to different bike types: e-bikes with a trailer, cargo-bikes or traditional bikes with a trailer, depending on the study group [49]. The intervention was successful in increasing the cycling frequency of the participants of the three intervention groups cycling to kindergarten and to work (cycling increase was around 1.5 days/week in autumn and spring) and a decreased car use was reported. Cycling behaviour to the grocery store did not change with the car being prevalent in this situation. Participants shared that appearing as a role model to their toddlers contributed to making cycling a desirable behaviour [50].

In a medical context, personalised, targeted education to active travel can be an effective way to increase walking and cycling levels. Prescribing physical activity sessions and active commuting to abdominally obese women over an 18-month randomized clinical trial was successful in achieving a 34% reduction in car commuting [114].

#### Additional Physical Activity

Contrary to the “ActivityStat” hypothesis stating that an increase in physical activity in one domain will be compensated by a decrease in another one, there appears to be a positive relationship between active travel and physical activity [115]. The British study making this claim recorded that each percentage point increase in (non-school) active travel led to an additional 0.38 increase in MVPA. In other studies, aside from physical activity inherent to the travel itself, walking and or cycling were associated with high engagement in either moderate, vigorous, or overall physical activity. The exact combination of associations seemed to be sex-dependent in European children and adolescents [82–85].

#### Car Culture and Active Travel

Overall, motorized transportation (not including public transport) is negatively associated with active travel. Traffic noise and parking space for cars are inversely correlated with walking [13]. Presence of a main road to school or having a parking space at work are example of car-related adaptations of the built environment that are negatively associated with active travel behaviour [odds ratio of walking, respectively cycling for commuting if individuals have a parking space at work (95% C.I): 0.53 (0.50–0.57) resp. 0.77 (0.68–0.86)] [40, 57]. Similarly, access to motorbikes or cars was negatively related to the usage of the Spanish bike sharing system mentioned earlier [28]. Furthermore, the presence of traffic or car parking near one’s home negatively impacts the children’s perception that the local place is a safe place for them to play outside or to walk alone after dark [odds ratio for qualifying the local area as a good place to grow if the nearby road was full of parked cars (95% C.I): 0.81 (0.76–0.85)] [58]. Integration of a reflection on car mobility during the process of obtaining a driving license (in form of a one hour lesson on active transportation) made future drivers significantly more aware of car-sharing schemes but failed at increasing the intention to use active modes of transportation [116].

On the other hand, punctual public transportation and stations within walking distance were positively associated with walking [13]. In cross-sectional studies, having a subscription to the public transport service correlated with walking for commuting (odds ratio: 4.06, 95%CI.: 3.78–4.35) [57]. Public and active transport thereby appear as complementary to active modes of transportation.

#### Socio-Economic Environment and Active Travel

Taking into account the social and economic situation of the population or individuals is important for understanding the additional mechanisms underlying travel behaviour. Regarding social relations, such as the interactions with others (acquaintances and strangers) and perceived community support, crowded spaces, and a sense of abandonment were negatively correlated with active travel in adults [13]. A cross-sectional study reported that adults scoring poorly on psychosocial attributes, (which they define as perceived social support, perceived barriers and self-efficacy) are the ones that respond most positively to mobility infrastructure interventions with increased walking for recreation and leisure-time physical activities [17].

Parents perceiving social pressure to walk with their kids engaged more in active travel [13]. Furthermore, when the children felt their parents had a negative perception of the environment, they showed a preference for car travel to school [48]. However, if parents displayed a physically active lifestyle and effective support to their children, the later were more likely to engage in physical activity [95, 139].

Regarding the effect of the wealth of the household, possessing one or more vehicle was negatively related to active travel to school [86]. The same study reported that children living in deprived areas of high-income countries showed a positive association with active travel to school, despite safety concerns. The authors report that this behaviour could be the result of a financial necessity rather than of a deliberate choice.

### Health Outcomes

Physical activity deriving from active travel and reduction of sedentary behaviour is highly correlated with positive health outcomes. The effects most often evaluated together with active mobility behaviour are cardio and respiratory health, obesity, musculoskeletal health, and mental health. An overview of the results is displayed in Table 3 with detailed results presented in a table in Supplementary Appendix S3.

**TABLE 3:** Summary of findings linking mobility and health outcomes (Switzerland, 2024).

Outcome	Summary of findings (associations)	References
Cardiovascular and/or respiratory health *assessed by measures of physiological parameters or incidence of particular conditions*	Decreased incidence of coronary heart disease, stroke, hypertension, cholesterol, and cardiovascular mortality with active commuting and cycling	(96–98, 100, 117)
	Increased respiratory fitness (greater value of maximal volume of oxygen) with active commuting	(97)
	Improved cardiorespiratory outcomes with walking, e-cycling, and cycling without assistance (dose-dependent)	(83, 89, 101, 106, 119, 123–125)
	Decreased incidence of hypertension with cycling (dose-dependent)	(118)
	Overall decrease of cardiorespiratory health with increased use of motorized transportation	(83, 99)
	Increased exposure to pollution with non-motorized commuting	(61, 65–68)
	High increase of mortality with transport’s pollution	(75)
	Protective effect of physical activity, exceeding the health prejudice of pollution exposure and traffic accidents	(62, 69)
Obesity *assessed using the body mass index (BMI), body fat percentage or waist circumference*	Active travel associated with lower BMI	(57, 87, 98, 102–104, 120, 121)
	Limited evidence for interventions promoting physical activity or active travel on obesity outcomes	(101, 105, 114, 127)
Musculoskeletal health *assessed using the incidence of fractures or by measuring parameters measured during a physical exercice*	Decreased risk of fracture with increasing leisure-time physical activity	(122)
	Increased maximal cycling power, muscle endurance or flexibility with biking (in particular non-electrically assisted biking)	(88, 97, 123)
Mental health *assessed by questionnaires*	Improved health and happiness levels with increasing active travel	(47)
	Improved wellbeing and health-related quality of life with increased physical fitness and mode of commuting in children	(89)
	Improved mental health and overall health with active commuting	(110)

### Co-Benefits

When looking at the contribution of the positive and negative health outcomes of active transport on the population, there is on one side: protective effect against cardiovascular and respiratory condition, type II diabetes, hypercholesterolemia, and reduced obesity; and on the other side: traffic accidents and air pollution exposure. At the individual level, the person engaged in the active behaviour has been showed to face a significant net beneficial effect [61, 62]. The gain in life-years due to adoption of daily cycling habits was evaluated as nine times greater than the years of life lost due to increased exposure to pollution [63]. The modal shift has also been reported to be clearly beneficial at the community level as well due to overall decrease in fuel-burning related pollution and noise reduction. Studies have forecast net avoided costs for the NHS amounting to £6 billion within a 20-year period [64] and for the Stockholm county’s healthcare budget (the net benefit amounted to 8.7% of the initial investments on infrastructures, which were of 900€ per year per person shifting from cars to bikes) [142].

Car prevalence in the overall urban space has been associated with detrimental perception of the environment, e.g., a lack of safety and community feeling. Contrastingly, active modes of transportation have been found to favour social interaction and the freed space could be allocated to other types of infrastructures supporting social gatherings such as Play Streets [55] or greenspaces.

Last but not least, a modal shift from car driving to walking or biking would significantly reduce GHG emissions, thus directly contributing to climate change mitigation. In a modelisation of a widespread adoption of e-bikes for commute trips across the Swiss population, a GHG emission reduction up to 10% of national fossil fuel-based emissions was estimated [143]. At the scale of the city of Barcelona, the implementation of bike sharing stations was estimated to have avoided the emission of 9,000 tons of CO_2_ from fossil fuel-based vehicles [61]. Finally, a modal shift would reduce at the source the microplastic pollution of water and soils originating from the friction of car tires with the brakes and road [144].

## Discussion

**FIGURE 2:**
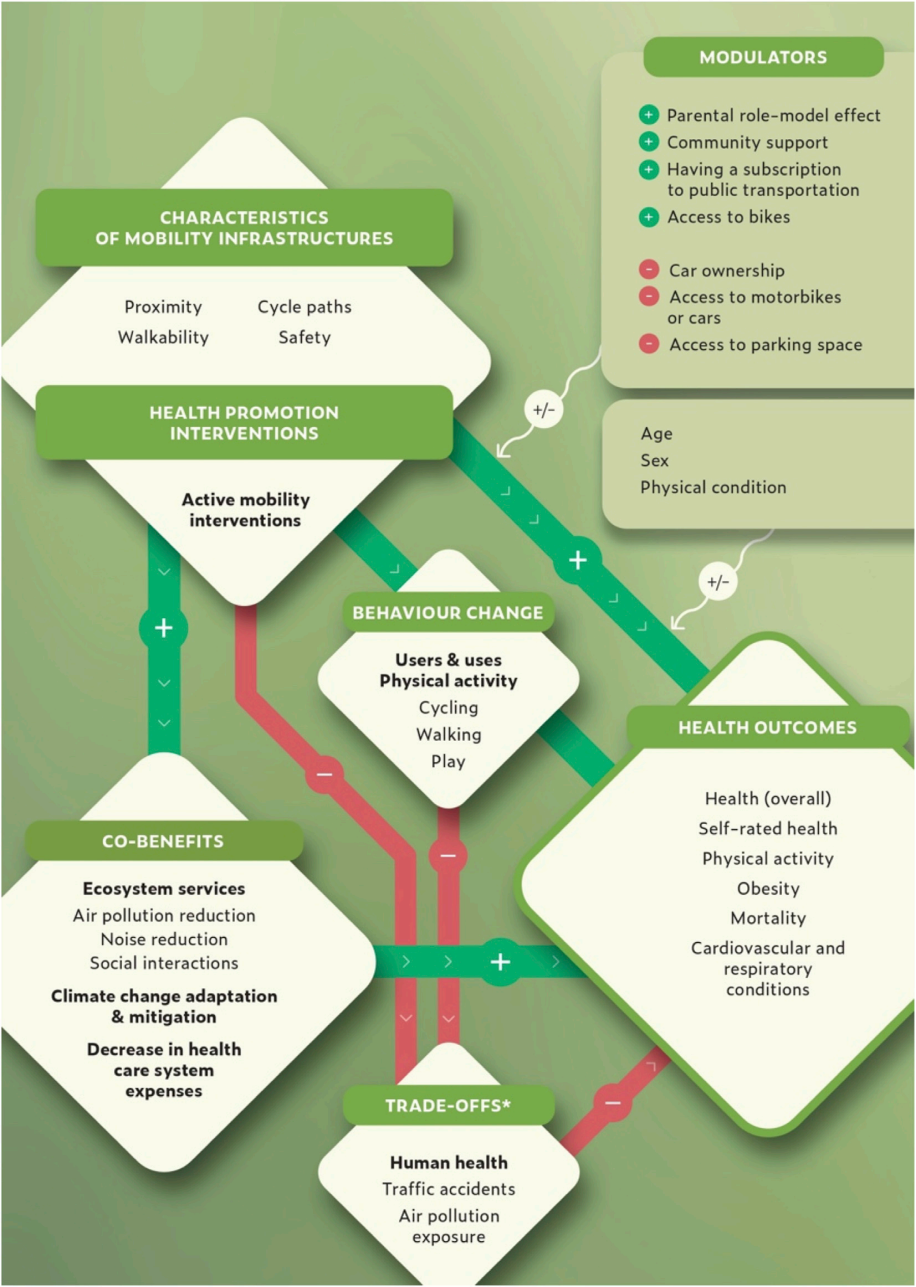
Main relationships between mobility infrastructures, behaviour change, health outcomes and co-benefits (Switzerland, 2024). *Specifically for cyclists but not for the overall population. Studies reported that some characteristics of mobility infrastructure (as well as interventions addressing active mobilities) lead to various positive health outcomes, notably via the induced physical activity. The amplitude of the positive effect is not homogenous across population groups and is subjected to modulators. Co-benefits, in terms of response to climate change, healthcare systems expenses, and improvement to the local environment also result from these mobility interventions, and in turn reinforce prevent from adverse health effects. On the other side, due to an increased exposure to traffic accidents and air pollution the positive health outcomes can be attenuated. The studies however report that the latter negative effect is marginal in relation to the other health benefits facilitated by active mobility.

This scoping review reports on features of the built environment and interventions related to active behaviour patterns, health outcomes and associated co-benefits. The identified links between these topics of interest are reported in Figure 2.

The literature demonstrates strong evidence that active transportation is positively associated with walkable environments [8–11, 113], though the contribution of each individual environmental features included in the term walkability is unclear [9, 10]. Cycling is favoured by the presence of dedicated cycle routes, and low traffic danger [22–24, 30, 36, 37, 81]. Addressing the issue of objective and perceived traffic danger allows a greater diversity of active travelers, and women cyclists in particular [23, 27]. Overall, despite quality of active mobility infrastructures, distance to destination remains a major barrier to active transportation [12, 22, 47, 48, 113, 141]. Educating the population about the benefits of active transportation [22, 50] and providing the opportunity to use bikes facilitated the recruitment of more people to active transportation [28, 49]. Contrastingly, owning a car and car prevalence is negatively associated with active transportation and affects children’s perception of the environment [13, 28, 40, 48, 57, 86].

Numerous cross-sectional studies report a significant association between active transportation and good physical condition. Better cardiac [96–100, 117, 118] and respiratory health [97, 101, 119] are reported in active travelers compared to passive ones. Similarly, positive associations are found between active mobility and reduced obesity [57, 87, 98, 102–104, 120, 121], lower risk of fracture [122], as well as better fitness [88, 97, 123] and mental health [47, 89, 102, 105]. Improved health effects were observed for biking compared to walking due to the higher energy expenditure profile of this activity [83, 89, 101, 106, 119, 123–125]. One major drawback that cyclists face is the increased exposure to traffic pollution compared to their passive counterparts [61, 65–68]. However, the health benefits of cycling outweigh this aspect. Indeed, a modal shift, from passive to active transportation, would have a marked advantage both at the individual level and at the broader society level in terms of noise reduction, particulate matter reduction, lower rate of traffic accidents and consequently reduced healthcare expenses [61–64, 69, 142–144].

According to these results, in order to increase active mobility among the population, mobility infrastructure should be revised to qualify as walkable areas, and cycling paths should be implemented. An effective transition implies decreasing car prevalence. Road space must be re-attributed to create distinct paths for pedestrians and for cyclists, both separated from car traffic. As biking, including electrically assisted biking, shows the greatest health benefits, the development of e-bike sharing networks are interesting solutions to be considered in supporting the large-scale modal shift. Car-free spaced would benefit social exchanges (for example, in the form of Plays Streets [55] and parks or gardens) and provide higher sense of security within the neighborhood. Urban planning must also carefully address proximity issues, by ensuring dense walking and cycling networks and ensuring that one’s home is at reasonable distance from essential services of daily-life (schools, grocery stores, etc.). Therefore, multimodality, between public and active transport should be facilitated for longer distances. However, to allow these recommendations to be implemented at a large scale in cities, strong commitment of political authorities is necessary, alongside a trans-sectorial approach [145, 146].

Overall this paper provides a usefull overview of the role of mobility infrastructure interventions to improve the health of the population while also increasing the quality of the environment at the local and at the European level. This article also gives indications of the non-infrastructural aspects that can contribute to, or impede, the adoption of active mobility. However, it must be noted that this review crosses several thematic fields (mobility, behaviour change, health and co-benefits) to highlight their interactions but that each discipline is vast and has many specificities not covered here. For example, behaviour change models, which would provide further insight into the complexity of adopting a new behaviour, were not addressed. Also a focus was placed on adopting active mobility and more marginally on limiting car use which is also a way of approaching the problem. Furthermore, the gender and socio-economic health disparities were only marginally addressed, but remain of major importance and must be taken into account in public policies and territorial planning [147]. Last, studies included in this review had a geographic focus on Europe and the results may not be representative of other regions.

Further research in form of longitudinal studies would be needed to see the long-term physical activity increase and health effects of infrastructure changes. In the meantime, modeling tools (e.g., the Health economic assessment - HEAT-tool) can help decision-makers to evaluate the averted health expenses due to increased walking and cycling. Such modelling studies and cost-analysis could greatly contribute to support the choice of the mobility scenario most adapted to a specific context, and the implementations of new policies for active transportation. Additionally, further research would be necessary to evaluate the effect of specific interventions such as school training (both theoretical and practical) over longer time scales, to see if the change in behaviour effectively persist after the end of the intervention period. Future studies reporting effects of before and after built environment changes would also be valuable as randomized control trials on infrastructural changes were scarce in this domain.

Finally, lowering GHG emissions is mandatory as part of climate change mitigation strategies against the warming climate as extensively underlined by the Intergovernmental Panel on Climate Change reports. Shifting away from cars and towards active modes of transportations would allow Switzerland to get a step closer to its reduction target (−50% GHG by 2030 compared to the level of 1990 [148]). On a broader scale, a modal shift is a win-win intervention to increase individual and community health, to decrease GHG emissions, and is documented as not only financially viable, but also advantageous for public health services.
