# Supplementary material for: Mobility Infrastructures and Health: Scoping Review of studies in Europe
Source: Public Health Rev. 2024 May 22;45:1606862. doi: 10.3389/phrs.2024.1606862 (PMC11150585; doi:10.3389/phrs.2024.1606862)
Supplement: Supplementary file 1 [file DataSheet1.docx]

**SUPPLEMENTARY MATERIAL:**

**Mobility Infrastructures and Health: Scoping Review
of Studies in Europe**

Michel, Sarah - Department of Family Medicine, Center for Primary Care and Public Health (Unisanté), University of Lausanne, Lausanne, Switzerland; sarah.michel@unil.ch

Banwell, Nicola – Interdisciplinary Centre for Research in Ethics (CIRE), University of Lausanne (UNIL), Lausanne, Switzerland; nicola.banwell@unil.ch

Senn, Nicolas – Department of Family Medicine, Center for Primary Care and Public Health (Unisanté), University of Lausanne, Lausanne, Switzerland; nicolas.senn@unisante.ch

## **Appendix S1 – Search strategy**

**Key search terms**

| **Topic** | **Key search terms** |
| --- | --- |
| *Review focus* | |
| 1. Mobility infrastructures with regards to active mobility | Active mobility, movement-friendly environment, exercise-friendly infrastructure, cycling, walking, sidewalk, footpath, cycling path, bike lane, bikeway, bike, bicycle, pedestrian zone, road, public transport, soft mobility, urban planning |
| *Outcome:* | |
| a) impacts on behavioural change | Mobility behaviour change, Health behaviour change, pro-environmental behaviour, health promoting behaviour, healthy behaviour, physical activity |
| b) impact on physical and mental health | Physical health, cardiovascular disease, type 2 diabetes, obesity, cancer, mental health, anxiety, depression, stress, hypertension, well-being, wellness, trauma, injury, pulmonary diseases, asthma, allergies (immune diseases), |
| c) environmental co-benefits relating to biodiversity and climate change | co-benefits, cobenefits, biodiversity, human nature contact, nature, climate change, greenhouse gas emissions, air pollution, soil pollution |

**Example of the search syntax used for Pubmed:**

(Bicycling[Mesh] OR Transportation[Mesh:NoExp] OR Pedestrians[Mesh] OR “Environment Design” [Mesh:NoExp] OR “Built Environment”[Mesh:NoExp] OR “City Planning” [Mesh] OR Urbanization[Mesh] OR "Active mobility"[tiab] OR "active commuting"[tiab] OR "active transport*"[tiab] OR "active travel"[tiab] OR bicycle*[tiab] OR bike*[tiab] OR cyclist*[tiab] OR "cycling path*"[tiab] OR "cycling facilit*"[tiab] OR "traffic calming"[tiab] OR "traffic safety"[tiab] OR "road safety"[tiab] OR "street safety"[tiab] OR footpath*[tiab] OR sidewalk*[tiab] OR "pedestrian friendly"[tiab] OR walkability[ti] OR "zebra cross*"[tiab] OR "street environment"[tiab] OR "Road environment"[tiab] OR "street connectivity"[tiab] OR "public transport*"[tiab] OR "soft mobility"[tiab] OR "urban planning"[tiab] OR "city planning"[tiab] OR "urban form*"[tiab] OR "urban design"[tiab] OR "built environment"[tiab] OR "urban environment"[tiab] OR "urban features"[tiab])

**AND**
(“Health behavior”[Mesh] OR ”health-related behavio*”[ti] OR “healthy behavio*”[ti] OR “behavior change*”[ti] OR “behaviour change*”[ti] OR “environmental awareness”[ti] OR  “health behavio*”[ti] OR “health promoting behavio*”[ti] OR “mobility behaviour change”[ti] OR “multiple behavio*”[ti] OR “multiple health behavio*”[ti] OR “pro-environmental behavio*”[ti] OR “proenvironmental behavio*”[ti] OR “Leisure Activities”[Mesh:NoExp] OR “leisure activit*”[ti] OR "Sports"[Mesh:NoExp] OR “sport*”[tiab] OR “running”[tiab] OR “walk”[tiab] OR “walking”[tiab] OR “smoking”[tiab] OR "social connect*"[tiab] OR "Social Cohesion"[Mesh] OR "social cohesion"[tiab] OR “Social skills”[Mesh] OR “social skills”[tiab] OR “prosocial behavio*”[tiab] OR “active lifestyle”[tiab] OR “Sleep”[Mesh] OR “sleep”[tiab] OR “Exercise”[Mesh] OR “exercise*”[ti] OR “physical activit*”[tiab] OR “Diet”[Mesh:NoExp] OR “diet”[tiab] OR “eating”[tiab] OR “nutrition”[tiab] OR "Feeding Behavior"[Mesh])

**AND**

("Europe"[Mesh] OR Europe*[tiab] OR Andorra[tiab] OR Austria*[tiab] OR Balkan[tiab] OR Belgium[tiab] OR Britain[tiab] OR Danish[tiab] OR Denmark[tiab] OR England[tiab] OR Finland[tiab] OR France[tiab] OR French[tiab] OR German*[tiab] OR Gibraltar[tiab] OR "United Kingdom"[tiab] OR Greece[tiab] OR Iceland[tiab] OR Ireland[tiab] OR Italy[tiab] OR Liechtenstein[tiab] OR Luxembourg[tiab] OR "Mediterranean Region"[tiab] OR Monaco[tiab] OR Netherlands[tiab] OR "Nordic Countries"[tiab] OR Norway[tiab] OR Portug*[tiab] OR "San Marino"[tiab] OR Scandinavia* OR Spain[tiab] OR Spanish[tiab] OR Sweden OR Swiss[tiab] OR Switzerland[tiab] OR Transcaucasia[tiab] OR Vatican[tiab])

**AND**

(clinicalstudy[Filter] OR clinicaltrial[Filter] OR comparativestudy[Filter] OR controlledclinicaltrial[Filter] OR governmentpublication[Filter] OR guideline[Filter] OR meta-analysis[Filter] OR observationalstudy[Filter] OR pragmaticclinicaltrial[Filter] OR randomizedcontrolledtrial[Filter] OR review[Filter] OR systematicreview[Filter])

**AND**

(English[la] OR French[la]) **AND** (2000:2023[pdat])

## **Appendix S2 – Inclusion and exclusion criteria**

| **Inclusion criteria** | **Exclusion criteria** |
| --- | --- |
| Peer-reviewed academic articles including:   - Research studies with various design, interventional and observational (e.g. randomised control trials, case-control studies, pre-post studies, observational studies) - Modelling studies linking to health and at least one of the two focus areas - Literature reviews articles of all types | Expert commentaries and non-empirical academic articles, case reports |
| Grey literature from reputable international organisations in relevant domains (for example WHO, IPCC, IUCN, UN Biodiv, IPBES, UN Habitat) | Modelling studies not linking to health |
| Policy recommendations and guidelines when available | Non-peer reviewed publications including conference proceedings. |
| Published since 2000 | Articles not written in French and English |
| Swtizerland (in particular for illustrative case studies and examples) and Europe |  |
| Focus of the literature is on behavioural change, health impacts and environmental co-benefits of mobility infrastructure and/or greenspaces and parks |  |

## **Appendix S3 – Overview of the studies from the literature search included in the review**

**Overview of the studies from the literature search included in the review, sorted by author**
**Age is provided when this information was available in the original article*

***The main results are, for most, direct quotes of the corresponding articles.*

| Author | Year | Article Name | Type of study | Population | Age* | Intervention | Outcome | Main results** |
| --- | --- | --- | --- | --- | --- | --- | --- | --- |
| Aittasalo | 2019 | Socio-Ecological Natural Experiment with Randomized Controlled Trial to Promote Active Commuting to Work: Process Evaluation, Behavioral Impacts, and Changes in the Use and Quality of Walking and Cycling Paths | Randomised control study | Adults |  | Built environment and promotion of active travel | Active travel | Increase in the employees’ motivation for active commuting to work but no impact on their actual behavior |
| Akinci | 2022 | How different are objective operationalizations of walkability for older adults compared to the general population? A systematic review | Systematic review | Older people, General population | older people : 65y+ | Built environment | Walking | Most of the papers included in the review found a positive association between walkability and walking-related outcomes for both groups: older people, and the general population. |
| Andersen | 2009 | Physical fitness in relation to transport to school in adolescents: the Danish youth and sports study | Cross sectional study | Youth (adolescents, teenagers) | 15-19y | Cycling | Physical fitness | Commuter bicycling may be a way to improve health in adolescents. |
| Andersen | 2022 | Socioeconomic position, built environment and physical activity among children and adolescents: a systematic review of mediating and moderating effects | Systematic review | Children; Youth (adolescents, teenagers) |  | Built environment | Physical activity | There was no evidence to support that the built environment functions as a mediator in the relationship between socioeconomic position and physical activity. |
| Aranda-Balboa | 2022 | The Effect of a School-Based Intervention on Children's Cycling Knowledge, Mode of Commuting and Perceived Barriers: A Randomized Controlled Trial | Randomised control study | Youth (adolescents, teenagers) |  | Promotion of active travel | Cycling, safety perception | No change in the rates of cycling and active mode of commuting to/from school at baseline and follow-up in both cycling and control groups. The cycling group increased the perception of built environment (walk) as a barrier to actively commute. |
| Avila-Palencia | 2017 | The relationship between bicycle commuting and perceived stress: A cross-sectional study | Cross sectional study | Adults |  | Cycling | Stress | Bicycle commuters had significantly lower risk of being stressed than non-bicycle commuters |
| Bassett | 2008 | Walking, cycling, and obesity rates in Europe, North America, and Australia | Cross sectional study | Mixed |  | Active travel | Obesity | European countries that rely heavily on walking and cycling have lower rates of obesity (as opposed to Canada, US and Australia) |
| Bere | 2009 | The association between cycling to school and being overweight in Rotterdam (The Netherlands) and Kristiansand (Norway) | Cross sectional study | Youth (adolescents, teenagers) |  | Cycling | Overweight | Cycling to school is clearly related to weight status in both the Rotterdam and the Kristiansand sample. |
| Berglund | 2016 | Active Traveling and Its Associations with Self-Rated Health, BMI and Physical Activity: A Comparative Study in the Adult Swedish Population | Cross sectional study | Adults | 45-75y | Active travel | Sealf-rated health, BMI | Significant difference in self-reported health between active and inactive travelers. Significant difference in self-reported BMI that is considered overweight or obese |
| Bjørnarå | 2019 | From cars to bikes - The effect of an intervention providing access to different bike types: A randomized controlled trial | Randomised control study | Adults |  | Access to bikes | Cycling | Providing parents with children in kindergarten with access to e-bikes might result in increased and sustained cycling, also during the winter season |
| Bjørnarå | 2020 | Cumbersome but desirable-Breaking the code of everyday cycling | Randomised control study | Adults |  | Access to bikes | Cycling | Despite challenging weather conditions, parents of young children may experience cycling as cumbersome but desirable and that bike access could contribute to increasing the feasibility of cycling everyday. |
| Blond | 2016 | Prospective Study of Bicycling and Risk of Coronary Heart Disease in Danish Men and Women | Prospective study | Older people |  | Cycling | Coronary heart disease incidence | Cycling and adopting a cycling behaviour are both associated with lower risk of coronary heart disease |
| Bluhm | 2011 | Cardiovascular effects of environmental noise: research in Sweden | Scoping review | Mixed |  | Traffic noise exposure | Cardiovascular health | Recent Swedish noise studies support the hypothesis of an association between residential exposure to road as well as aircraft traffic noise and hypertension. Increased risk has also been indicated for acute myocardial infarction in relation to road traffic noise. |
| Børrestad | 2011 | Seasonal and socio-demographic determinants of school commuting | Cross sectional study | Children |  | Season and proximity | Active travel | cycling was the dominant mode of commuting in fall and spring, and walking the dominant mode in winter. |
| Bunn | 2009 | Area-wide traffic calming for preventing traffic related injuries | Systematic review | Mixed |  | Built environment (area-wide traffic calming) | Road traffic death and injuries | Area-wide traffic calming in towns and cities may be a promising intervention for reducing the number of road traffic injuries and deaths |
| Chillón | 2010 | Active commuting to school in children and adolescents: an opportunity to increase physical activity and fitness | Cross sectional study | Children; Youth (adolescents, teenagers) |  | Active travel | Physical activity | Differences in moderate, vigorous, moderate to vigorous, and average physical activity levels were observed between active commuting to school (ACS) and non-ACS boys. No differences were observed in girls. |
| Chillón | 2011 | A systematic review of interventions for promoting active transportation to school | Systematic review | Children; Youth (adolescents, teenagers) | 6-18y | Built environment, promotion of active travel | Active travel | Interventions with appropriate school, parent, and community involvement and that work toward a specific goal (i.e., increasing active transportation) seemed to be more effective than interventions that were broader in focus |
| Chillón | 2011 | Active commuting and physical activity in adolescents from Europe: results from the HELENA study | Cross sectional study | Youth (adolescents, teenagers) | 12-18y | Active travel | Physical activity | positive association between active commuting and physical activity levels in adolescents and these associations were stronger in boys than in girls. Results also suggest that younger adolescents spend more time in active commuting than older adolescents. |
| Christiansen | 2013 | Effect of a school environment intervention on adolescent adiposity and physical fitness | Randomised control study | Youth (adolescents, teenagers) | 11-14y | Built environment | Adiposity, aerobic fitness, and musculo-skeletal strength | No significant difference in outcomes between intervention and comparison schools. |
| Colom | 2021 | Neighbourhood walkability and physical activity: moderating role of a physical activity intervention in overweight and obese older adults with metabolic syndrome | Randomised control study | Older people | 55-75y | Built environment and promotion of active travel | Physical activity | Higher walkability combined with a physical activity intervention could be the most effective strategy to increase physical activity among older adults |
| Coombes | 2013 | Is environmental setting associated with the intensity and duration of children's physical activity? Findings from the SPEEDY GPS study | Cross sectional study | Children | 9-10y | Built environment | Physical activity | In terms of activity intensity, buildings and roads and pavements were used relatively more for light activity. Roads and pavements also appear supportive of bouts of MVPA. |
| Cooper | 2005 | Physical activity levels of children who walk, cycle, or are driven to school | Cross sectional study | Children |  | Travel mode | Physical activity | Boys who walked or cycled to school were significantly more physically active than those traveling by car. In girls, walking but not cycling to school was significantly associated with higher daily physical activity levels |
| Costa | 2020 | A Scoping Review of Children and Adolescents' Active Travel in Ireland | Scoping review | Children; Youth (adolescents, teenagers) |  | (Built) environment | Active travel | The existing low levels of cycling to and from a destination, in conjunction with distance and safety barriers, are potentially contributing factors which might explain the difficulty in adopting active travel lifestyle behaviours for children and adolescents. |
| D'Haese | 2015 | Cross-continental comparison of the association between the physical environment and active transportation in children: a systematic review | Systematic review | Children | 6-12y | Built environment | Active travel and physical activity | An activity friendly neighborhood that is walkable, dense, accessible, equipped with walk/cycle facilities and safe from traffic is associated with more active transportation to school in children. |
| D'Haese | 2015 | Organizing "Play Streets" during school vacations can increase physical activity and decrease sedentary time in children | Randomised control study | Children |  | Play streets | Physical activity | The introduction of a Play Street is an effective intervention at neighborhood level to increase urban children’s moderate to vigorous physical activity and decrease sedentary time during summer vacations. |
| Dadpour | 2016 | Understanding the Influence of Environment on Adults' Walking Experiences: A Meta-Synthesis Study | Systematic review | Adults | 18-65y | (Built) environment | Appreciation of the walking experience | List of factors influencing positively or negatively the walking experience |
| DeBourdeaudhuij | 2003 | Environmental correlates of physical activity in a sample of Belgian adults | Cross sectional study | Adults | 18-65y | Built environment | Physical activity | Minutes of walking and of moderate-intensity activity were related to quality of sidewalks and accessibility of shopping and public transportation. Vigorous physical activity was related to presence of activity supplies in the home and number of convenient activity facilities outside the home |
| deBruijn | 2005 | Determinants of adolescent bicycle use for transportation and snacking behavior | Cross sectional study | Youth (adolescents, teenagers) |  | (Built) environment | Cycling | Direct association between bicycle use and the distal variables school type, ethnicity, and degree of urbanization |
| deNazelle | 2017 | Comparison of air pollution exposures in active vs. passive travel modes in European cities: A quantitative review | Miscelaneous type of review | Adults |  | Travel mode | Pollution exposure | Exposure is the greatest for car riders and the lowest for pedestrians. |
| Eichinger | 2018 | Subjectively and Objectively Assessed Behavioral, Social, and Physical Environmental Correlates of Sedentary Behavior in Preschoolers | Cross sectional study | Children | 6-8y | Social and (built) environment | Physical activity | Leisure-time sedentary behavior decreased with greater levels of moderate-to-vigorous physical activity (both weekends and weekday afternoons), participation in organized sports, parental leisure-time physical activity, as well as greater parental traffic safety perceptions (weekends only) |
| Flint | 2016 | Active commuting and obesity in mid-life: cross-sectional, observational evidence from UK Biobank | Cross sectional study | Adults | 40-69y | Active travel | Obesity | In fully adjusted models, compared with their car-only counterparts, mixed public and active transport commuters had significantly lower BMI, as did cycling or cycling and walking commuter |
| Foster | 2004 | Changing the environment to promote health-enhancing physical activity | Systematic review | Adults | 18y+ | Built environment | Health enhancing physical activity | Environmental change studies showed a small increase on HEPA behaviour, but the relative impact of environment changes was not evaluated |
| Fraser | 2010 | Cycling for transport and public health: a systematic review of the effect of the environment on cycling | Systematic review | Mixed |  | Built environment | Active travel (cycling) | The environmental factors identified as being positively associated with cycling included presence of dedicated cycle routes or paths, separation of cycling from other traffic, high population density, short trip distance, proximity of a cycle path or greenspace and for children projects promoting ‘safe routes to school’. |
| González | 2020 | Active school transport among children from Canada, Colombia, Finland, South Africa, and the United States: A tale of two journeys | Cross sectional study | Children | 9-11y | Proximity and car ownership | Active travel | Distance to school is a consistent correlate of active travel to school in different contexts. Vehicle ownership were associated with a lower likelihood of engaging in active travel to school in sites in upper-middle- and high-income countries |
| Goodman | 2011 | Activity compensation and activity synergy in British 8-13 year olds | Cross sectional study | Children | 8-13y | Active travel | Physical activity | Activity synergy between active travel and physical activity at other times |
| Goodman | 2013 | Effectiveness and equity impacts of town-wide cycling initiatives in England: a longitudinal, controlled natural experimental study | Cross sectional study | Mixed |  | Built environment | Cycling | Cycling to work has increased (and driving to work decreased) in the intervention towns |
| Götschi | 2015 | Contrasts in active transport behaviour across four countries: how do they translate into public health benefits? | Modelling study | Mixed | 15y+ | Active travel | Health benefits (premature deaths and DALY) | A substantial amount of premature deaths and DALY could be avoided if England and Wales adopted the travel patterns for walking and cycling of Switzerland or the Nederlands. |
| Gram | 2017 | Anti-inflammatory effects of active commuting and leisure time exercise in overweight and obese women and men: A randomized controlled trial | Randomised control study | Adults | 20-45y | Active travel | Anti-inflammatory effect | Anti-inflammatory effect of active commuting and moderate, but not vigorous, intensity leisure time exercise |
| Hajna | 2015 | Associations between neighbourhood walkability and daily steps in adults: a systematic review and meta-analysis | Systematic review | Adults | 18y+ | Built environment | Walking (steps per day) | living in high compared to low walkable neighbourhoods is associated with accumulating766 more steps per day |
| Hamer | 2008 | Active commuting and cardiovascular risk: a meta-analytic review | Systematic review |  |  | Active travel | Cardiovascular risk (mortality, incident coronary heart disease, stroke, hypertension and diabetes) | The overall meta-analysis demonstrated a robust protective effect of active commuting on cardiovascular outcomes |
| Hamilton | 2004 | Cycling: The risks | Narrative review | Mixed |  | Road safety | Cycling | Cyclists stand to gain more from road safety than any other road user. |
| Harrison | 2012 | A framework for understanding school based physical environmental influences on childhood obesity | Scoping review | Children; Youth (adolescents, teenagers) | < 18y | Built environment, school ground | Active travel, physical activity | The likelihood of active travel is affected by the provision for walking and cycling to school. Playground size, design and equipment is associated with physical activity |
| Hartog | 2011 | Do the health benefits of cycling outweigh the risks? | Modelling study | Adults | 18-64y | Active travel | Health costs and benefits | The estimated beneficial effect on all-cause mortality rates of the increased physical activity due to cycling is substantially larger than the potential mortality effect of increased inhaled air pollution doses and the effect on traffic accidents. |
| Hemmingsen | 2015 | Controlled exposure to particulate matter from urban street air is associated with decreased vasodilation and heart rate variability in overweight and older adults | Randomised control study | Adults; Older people | 55-83y | Pollution | Vasodilation and heart rate variability | Exposure to real-life levels of particulate matter from urban street air impairs the vasomotor function and heart rate variability in overweight middle-aged and elderly adults. |
| Hemmingsson | 2009 | Increased physical activity in abdominally obese women through support for changed commuting habits: a randomized clinical trial | Randomised control study | Adults | 30-60y | Promotion of active travel | Active travel and body weight | Added support (physical activity prescription, bicycle..) had a positive effect on the achievement of cycling goals. Body weight did not change between groups. |
| Hemmingsson | 2011 | Bicycling but not walking is independently associated with fasting insulin in abdominally obese women | Randomised control study | Adults; Women | 30-60y | Active travel | Insuline production | Bicycling to and from work may be more important than walking for reducing hyperinsulinemia |
| Höchsmann | 2018 | Effect of E-Bike Versus Bike Commuting on Cardiorespiratory Fitness in Overweight Adults: A 4-Week Randomized Pilot Study | Randomised control study | Adults | 18-50y | Cycling | Cardiorespiratory fitness | E-bikes may have the potential to improve cardiorespiratory fitness similar to conventional bicycles despite the available power assist |
| Hollingworth | 2015 | Dose-response associations between cycling activity and risk of hypertension in regular cyclists: The UK Cycling for Health Study | Cross sectional study | Mixed | 16-88y | Cycling | Risk of hypertension | Inverse, dose–response relationship between cycling volume and risk of diagnosed hypertension |
| Jacob | 2021 | Economics of Interventions to Increase Active Travel to School: A Community Guide Systematic Review | Systematic review | Mixed | students and parents | Built environment | active travel, economical cost-benefit ratio | Interventions that improve infrastructure and enhance the safety and ease of active travel to schools generate societal economic benefits that exceed the societal cost |
| Jarrett | 2012 | Effect of increasing active travel in urban England and Wales on costs to the National Health Service | Modelling study | Mixed |  | Active travel | Healthcare costs | increased walking and cycling in urban England and Wales could induce a substantial reduction in the potential effect on the NHS budget |
| Jørgensen | 2013 | Population-level changes to promote cardiovascular health | Scoping review | Mixed |  | Built environment | Active travel | Changes in national policies and the built environment will integrate physical activity into daily life |
| Juhra | 2012 | Bicycle accidents - Do we only see the tip of the iceberg?: A prospective multi-centre study in a large German city combining medical and police data | Prospective study | Mixed |  | Active travel | Cycling accidents | Bicycle accidents occur more frequently than indicated by police records. Elderlies and children are more prone to falling by their own while young adults’ accidents more often involve collisions with motorized vehicles. |
| Karanasiou | 2014 | Assessment of personal exposure to particulate air pollution during commuting in European cities-Recommendations and policy implications | Scoping review |  |  | Travel mode | Personal exposure to particulate matter | Personal exposure to PM for bus and car commuters depends on several parameters including traffic intensity. |
| Kelly | 2014 | Systematic review and meta-analysis of reduction in all-cause mortality from walking and cycling and shape of dose response relationship | Systematic review | Mixed |  | Walking, cycling | All-Cause mortality | Walking and cycling were shown to reduce the risk of all-cause mortality, adjusted for other PA. |
| Kirk | 2013 | How to get more people with diabetes cycling | Narrative review | Adults |  | Social support | Cycling | Cycling groups are interesting levers for health professionals to encourage patient with diabetes to engage in physical activity |
| Krenn | 2014 | Route choices of transport bicyclists: a comparison of actually used and shortest routes | Cohort study | Adults |  | Cycling environment | Cycling detour | Bicyclists used bicycle pathways/lanes, flat roads, and attractive areas rather than the shortest possible routes. |
| Lamb | 2013 | Cycling as a mode of transport: A possible solution for the increasing burden of type 2 diabetes? | Scoping review |  |  | Cycling | Risk of type II diabetes | Cohort studies performed in several European countries have shown active travel to reduce the risk of developing T2DM and to reduce all-cause and cardiovascular mortality among individuals with T2DM |
| Langlois | 2017 | Association of socioeconomic, school-related and family factors and physical activity and sedentary behaviour among adolescents: multilevel analysis of the PRALIMAP trial inclusion data | Cross sectional study | Youth (adolescents, teenagers) | 14-18y | Socioeconomic and family variables | Physical activity | Both girls and boys with parents who had a high physical activity level practiced more total physical activity than did other adolescents. For both boys and girls active commuting was associated with residence area. |
| Liao | 2020 | How Does Walkability Change Behavior? A Comparison between Different Age Groups in the Netherlands | Cross sectional study | Mixed |  | Walkability | out-of-home activities | Absence of a relationship between walkability and out-of-home activities in the children group and the elderly group |
| Lu | 2014 | Perceived barriers to children's active commuting to school: a systematic review of empirical, methodological and theoretical evidence | Systematic review | Mixed | 4-19y and related adults | Perceived barriers of the (built) environment | Active travel | For middle school students, the identified perceived barriers were mostly about physical environmental characteristics, including distance, traffic safety, bad weather, and lack of sidewalks. In contrast, perceived barriers for elementary school children included various personal, social environmental, and physical environmental characteristics. |
| Mäki-Opas | 2014 | The association between physical environment and cycling to school among Turkish and Moroccan adolescents in Amsterdam | Cross sectional study | Children, Youth (adolescents, teenagers); | 10-18y | Cycling infrastructure | Cycling | Bicycle-friendly infrastructure and an enjoyable environment were not important factors for cycling to and from school among those with no cultural cycling background. |
| Manigrasso | 2017 | Pedestrians in Traffic Environments: Ultrafine Particle Respiratory Doses | Modelling study | Adults; Persons living with disabilities |  | Traffic environment | Deposited particles in the respiratory tract | On workdays, ultrafine particle number concentrations are much higher due to the strong contribution of vehicular exhausts compared to weekdays. |
| Masoumi | 2017 | Associations of built environment and children's physical activity: a narrative review | Narrative review | Children | 3-12y | Proximity and parental support | Active travel | Distance to school is a key determinant for choosing active transportation to school. Lack of parental support towards active transportation leads to a preference in being driven to school. |
| Materová | 2022 | Surveillance of physical activity and sedentary behaviour in czech children and adolescents: a scoping review of the literature from the past two decades | Scoping review | Children; Youth (adolescents, teenagers) | 6-20y | Built environment, promotion of active travel | Active travel | Living within a 20-minute walking distance to school, a place of residence being in the same municipality as the school, high-walkable areas, and attending schools with policies and programmes promoting active transportation were positively associated with active commuting to school. |
| McVicar | 2022 | Systematic review and meta-analysis evaluating the effects electric bikes have on physiological parameters | Systematic review | Mixed |  | E-cycling, conventional cycling | energy expenditure, heartrate, oxygenuptake, power output and metabolic equivalents | E-cycling was associated with an increase in physiological response that can confer health benefits (sufficient to meet PA recommendations) |
| Menai | 2015 | Walking and cycling for commuting, leisure and errands: relations with individual characteristics and leisure-time physical activity in a cross-sectional survey (the ACTI-Cités project) | Cross sectional study | Adults |  | Walking and cycling | Active travel | Combinations of association exist between walking/cycling for commuting and walking/cycling for leisure and errands |
| Molina-García | 2015 | Bicycling to university: evaluation of a bicycle-sharing program in Spain | Cross sectional study | Adults |  | Promotion of a public bike sharing program | Cycling | Significant increase in the cycling rate during the program. Persistence of the behavior at follow-up for 20% of the participants. |
| Møller | 2011 | The effect on cardiorespiratory fitness after an 8-week period of commuter cycling--a randomized controlled study in adults | Randomised control study | Adults |  | Cycling | Cardiorespiratory fitness, body fat | After 8 weeks VO2max and cardio-respiratory fitness were significantly improved in the cycling group when compared to the control group and body fat was reduced. |
| Mueller | 2015 | Health impact assessment of active transportation: A systematic review | Systematic review | Mixed |  | Active travel, noise, pollution, traffic incident | Health benefits | Effects of increased PA contributed the most to estimated health benefits, which strongly outweighed detrimental effects of traffic incidents and air pollution exposure on health. |
| Mueller | 2021 | Integrating health indicators into urban and transport planning: A narrative literature review and participatory process | Narrative review | Mixed |  | Built environment | Health indicators | Ten planning principles and corresponding indicators to be used by urban and transport planners to ensure favorable health and well-being outcomes in cities |
| Mullan | 2003 | Do you think that your local area is a good place for young people to grow up? The effects of traffic and car parking on young people's views | Cross sectional study | Children; Youth (adolescents, teenagers) | 11-16y | Built environment | Safety | The current dominance of the motor vehicle on residential roads has greatly reduced the attractiveness of, and quality of, life in both urban and rural communities for young people in Wales |
| Murtagh | 2011 | Active travel to school and physical activity levels of Irish primary schoolchildren | Cross sectional study | Children | 9-11y | Active travel | Step-count | Children who walked or cycled to school had higher daily step counts than those who traveled by passive modes |
| Neumeier | 2020 | Effects of active commuting on health-related quality of life and sickness-related absence | Randomised control study | Adults |  | Active travel | Health-related quality of life | active commuting is able to improve the health related quality of life of working adults. |
| Nowicka | 2007 | Physical activity-key issues in treatment of childhood obesity | Rapid review | Children |  | Physical activity and active travel programs | Physical activity | It is essential to involve family and friends and to formulate realistic goals for long-term success |
| Nyhan | 2014 | Comparison of particulate matter dose and acute heart rate variability response in cyclists, pedestrians, bus and train passengers | Cohort study | Adults | 18-35y | Travel mode, pollution | Heart rate variability response | Exercise whilst commuting has an influence on inhaled particulate matter (PM) and PM lung deposited dose, and these were significantly associated with acute declines in heart rate variability, especially in pedestrians and cyclists. |
| O'Donoghue | 2007 | Exposure to hydrocarbon concentrations while commuting or exercising in Dublin | Modelling study |  |  | Travel mode, pollution | Inhaled pollutants | Cyclist inhaled a slightly greater mass of pollutants per journey than the bus passenger when breathing rate is taken into account |
| Oja | 2011 | Health benefits of cycling: a systematic review | Systematic review | Mixed |  | Cycling | fitness, cardiovascularrisk factors, all-cause mortality, cor-onary heart disease morbidity and mortality, cancer risk,and overweight and obesity | The strength of this evidence was strong for fitness benefits, moderate for benefits in cardiovascular risk factors, and inconclusive for all-cause mortality, coronary heart disease morbidity and mortality, cancer risk, and overweight and obesity |
| Ooms | 2017 | The Start2Bike program is effective in increasing health-enhancing physical activity: a controlled study | Cohort study | Adults |  | Promotion of cycling (programm for beginners) | Physical activity, Cycling | The program positively influences HEPA levels of participants by increasing participation in sport. At a 6 months follow-up a majority of the participants were still practicing cycling. |
| Panter | 2018 | Using alternatives to the car and risk of all-cause, cardiovascular and cancer mortality | Prospective study | Adults | 37-73y | Active travel | Morbidity and mortality | The general pattern of the results indicates that, irrespective of other physical activity, more active patterns of travel, compared with exclusive car use, were associated with reductions in risk of incident and fatal cardiovascular diseases and all-cause mortality. |
| Pucher | 2003 | Promoting safe walking and cycling to improve public health: lessons from The Netherlands and Germany | Cross sectional study | Mixed |  | Traffic regulations | Traffic accidents | Traffic regulations that strongly favor pedestrians and bicyclists have enabled lower fatality and injury rates in The Netherlands and Germany |
| Pucher | 2010 | Walking and cycling to health: a comparative analysis of city, state, and international data | Cross sectional study | Mixed |  | Active travel | Obesity | Significant negative relationships between active travel and self-reported obesity |
| Puggina |  | Policy determinants of physical activity across the life course: a 'DEDIPAC' umbrella systematic literature review | Umbrella review | Mixed |  | Built environment | Physical activity | At the population level, community- and street-scale urban design and land use policies were found to positively support physical activity levels, but levels of evidence were low |
| Quam | 2017 | Assessing Greenhouse Gas Emissions and Health Co-Benefits: A Structured Review of Lifestyle-Related Climate Change Mitigation Strategies | Miscelaneous type of review | Mixed |  | Active travel | Health benefits | Reviewed active transport articles concluded that their scenarios would reduce GHGE and increase physical activity resulting in net positive health outcomes |
| Ragettli | 2014 | Simulation of population-based commuter exposure to NO₂ using different air pollution models | Modelling study |  |  | Travel mode, pollution | Exposure to traffic-related air pollution | Highest median cumulative exposures were calculated along motorized transport and bicycle routes, and the lowest for walking. |
| Reich | 2020 | Effects of active commuting on cardiovascular risk factors: GISMO-a randomized controlled feasibility study | Randomised control study | Adults | 18-75y | Active travel | Physical activity | Actively covered distances through commuting significantly differed between groups |
| Renzi | 2022 | A nationwide study of air pollution from particulate matter and daily hospitalizations for respiratory diseases in Italy | Cross sectional study | Mixed |  | Pollution | Respiratory health | Evidence of harmful effect of PM 10 and PM2.5 on respiratory hospitalizations in Italy during 2006–2015 and positive association for a subgroup of respiratory out-comes such as asthma |
| Ribeiro | 2016 | The influence of socioeconomic, biogeophysical and built environment on old-age survival in a Southern European city | Cross sectional study | Older people | 75-94y | Built environment | Old-age survival | Walkability was unrelated to old-age survival |
| Rojas-Rueda | 2011 | The health risks and benefits of cycling in urban environments compared with car use: health impact assessment study | Modelling study | Mixed |  | Travel mode | Co-benefits | The health cost-benefit ratio of cycling instead of riding by car is largely in favor of cycling |
| Rojas-Rueda | 2021 | Environmental risk factors and health: An umbrella review of meta-analyses | Umbrella review | Mixed |  | Pollution and noise exposure | Health outcomes | This umbrella review identified 68 environmental exposures that were associated to 83 health outcomes. |
| Rowland | 2003 | Randomised controlled trial of site specific advice on school travel patterns | Randomised control study | Children |  | Advice on school travel patterns | Active travel and parental concern | No changes in active travel among children. Modest reduction in parental concern about traffic danger as a result of the intervention |
| Ruiz-Hermosa | 2018 | No Association Between Active Commuting to School, Adiposity, Fitness, and Cognition in Spanish Children: The MOVI-KIDS Study | Cross sectional study | Children | 4-7y | Active travel | Adiposity, physical fitness, and cognition | No differences in adiposity, physical fitness, and cognitive performance between active commuters and nonactive commuters. |
| Sallis | 2012 | Role of built environments in physical activity, obesity, and cardiovascular disease | Narrative review |  |  | Built environment | Active travel | Proximity and land use are key features to promote active travel and physical activity. Built environment interventions must be supported by changes in the social support and norms. |
| Sareban | 2020 | Effects of active commuting to work for 12 months on cardiovascular risk factors and body composition | Randomised control study | Adults |  | Active travel | Cardiovascular risk factors and body composition | No significant dose‐response relationship between active commuting and body composition could be observed. Neither for cardiovascular risk factors and active commuting. |
| Saucedo-Araujo | 2021 | Is children’s health-related quality of life associated with physical fitness and mode of commuting? PREVIENE Project | Cross sectional study | Children |  | Active travel | Health related quality of life and cardiorespiratory fitness | Scores for the total scores of health related quality of life were higher for children engaged in active travel. Boys who walked to school presented better cardiorespiratory fitness than their passive counterparts. |
| Schäfer | 2020 | Health effects of active commuting to work: The available evidence before GISMO | Systematic review | Adults | 18y + | Active travel | Maximal volume of oxygen, maximal power, diastolic blood pressure | the main results indicate that cycling and walk-ing to work at a self-paced intensity have a positive impact on indexes of fitness and health parameters. |
| Schnohr | 2012 | Intensity versus duration of cycling, impact on all-cause and coronary heart disease mortality: the Copenhagen City Heart Study | Cross sectional study | Adults | 21-90y | Cycling | all-cause and coronary heart disease mortality | Significant inverse association between cycling intensity and risk of all-cause and coronary heart disease death, but only a weak association with cycling duration. |
| Schüle | 2015 | Interactive and independent associations between the socioeconomic and objective built environment on the neighbourhood level and individual health: A systematic review of multilevel studies | Systematic review | Mixed |  | Built environment | Physical activity, weight/obesity | Objective built environmental metrics indicating higherwalkability were often associated with measures of higher individual physical activity independent from neighbourhood socio-economic position and individual factors. |
| Schulz | 2018 | Built environment and health: a systematic review of studies in Germany | Systematic review | Mixed |  | Built environment, noise and air pollution | Physical activity, wlaking, weight changes, blood pressure | The most convincing evidence existed on the relationships between destination proximity and street network and physical activity/walking. |
| Scotini | 2017 | Supporting active mobility and green jobs through the promotion of cycling | Cross sectional study |  |  | Policy measures on transportation | Economy (cycling jobs) | Amount of cycling related jobs could be increased if the cycling share of other European country would be increased to the level of Copenhaguen. |
| Sehlstedt | 2010 | Airway inflammatory response to diesel exhaust generated at urban cycle running conditions | Cohort study | Adults | 21-40y | Pollution | Airway inflammation | Diesel exhaust generated in by engines in urban running conditions induces an airway inflammatory response. |
| Shephard | 2008 | Is active commuting the answer to population health? | Rapid review | Mixed |  | Active commuting | Cardiovascular health | Active commuting has the potential to generate the 4 MJ weekly volume of physical activity commonly associated with enhanced health. In the case of cycling, the intensity also appears to fall into the cardio-respiratory training zone. The usual intensity of walking may be insufficient to benefit the cardiovascular health of fit young adults, although some adjustments are possible by adoption of a rapid pace. |
| Simons | 2009 | Electrically assisted cycling: a new mode for meeting physical activity guidelines? | Cohort study | Adults |  | (E-) cycling | Cycling intensity and speed, power output and heart rate | Intensity of cycling on an electrically-assisted, was sufficiently high to contribute to the moderate-intensity standard of the physical activity guidelines for adults but not to the vigourous intensity standard |
| Solomon | 2013 | Personal, social, and environmental correlates of physical activity in adults living in rural south-west England: a cross-sectional analysis | Cross sectional study | Adults | 18y + | (Built) environment | Physical activity | Inconvenience of public transport, and using recreational facilities outside the local village were associated with greater reported leisure-time physical activity. None of the village-level factors were associated with physical activity |
| Spence | 2020 | Potential Impact of Autonomous Vehicles on Movement Behavior: A Scoping Review | Scoping review | Mixed |  | Autonomous vehicles | Active travel, sedentarity | Autonomous vehicles will impact aspects of mode choice and the built environment of people residing in much of the developed world, resulting in reduced walking and more sitting |
| Stattin | 2017 | Leisure-Time Physical Activity and Risk of Fracture: A Cohort Study of 66,940 Men and Women | Cohort study | Adults |  | Active transport, physical activity | Risk of fracture | Walking/bicycling and weekly exercise sessions decreased the rate of all fractures |
| Stewart | 2015 | What interventions increase commuter cycling? A systematic review | Systematic review | Adults | 18y + | Built environment | Physical activitity, Active transportation | Evidence of what interventions will increase commuter cycling in low cycling prevalence nations is sparse. Wider environmental interventions that make cycling conducive appear to reach out to hard to define but larger populations. |
| Sugiyama | 2014 | Perceived neighbourhood environmental attributes associated with adults׳ recreational walking: IPEN Adult study in 12 countries | Cross sectional study | Adults | 18y-66y | (Built) environment | Walking | Perceived residential density, land use mix, street connectivity, aesthetics, safety from crime, and proximity to parks were linearly associated with recreational walking, |
| Tainio | 2021 | Air pollution, physical activity and health: A mapping review of the evidence | Miscelaneous type of review | Adults |  | Pollution exposure | physical activity | Air pollution may decrease physical activity levels during high air pollution episodes or may prevent people from engaging in physical activity overall in highly polluted environments |
| Thomas | 2013 | The safety of urban cycle tracks: a review of the literature | Miscelaneous type of review | Mixed |  | Cycling infrastructure | Collision and injuries | One-way cycle tracks are generally safer than two-way. When effective intersection treatments are employed, constructing cycle tracks reduces collisions and injuries. |
| Townshend | 2009 | Obesogenic urban form: theory, policy and practice | Miscelaneous type of review | Mixed |  | Built environment | Physical activity | higher residential densities, good levels of connectivity between streets, greater levels of land-use mix, pavement provision and areas that are perceived to be aesthetically pleasing and safe to be in enable and/or encourage people to lead more healthy, active lifestyles |
| Travert | 2019 | Built Environment and Health Behaviors: Deconstructing the Black Box of Interactions-A Review of Reviews | Scoping review | Mixed |  | Built environment | Health | Conceptual model helping the development of multi-component intervention strategies on the built environment to target health outcomes. |
| Tremblay | 2016 | Global Matrix 2.0: Report Card Grades on the Physical Activity of Children and Youth Comparing 38 Countries | Cross sectional study | Children; Youth (adolescents, teenagers) |  | (Built) environment | Physical activity | Contrarily to low income countries, higher income countries report better grades in infrastructure but lower levels of physical activity and higher levels of sedentarity. |
| VanCauwenberg | 2013 | Older adults' transportation walking: a cross-sectional study on the cumulative influence of physical environmental factors | Cross sectional study | Older people |  | (Built) environment | Walking | Distance is the major factor determing walking for transportation. Other environmental factors favor walking but for moderate distamces only. |
| VanDyck | 2014 | Interacting psychosocial and environmental correlates of leisure-time physical activity: a three-country study | Cross sectional study | Adults | 20-65y | Walkability | Physical activity | Perceived barriers to physical activity, perceived benefits of physical activity, social support from family and friends moderate the relationships of specific perceived environmental characteristics with walking for recreation and/or leisure-time moderate-to-vigorous physical activity |
| VanHolle | 2012 | Relationship between the physical environment and different domains of physical activity in European adults: a systematic review | Systematic review | Adults | 18-65y | Built environment (walking/cycling infrastructure) | Total physical activity, walking, cycling | Convincing evidence for a positive relationship with total PA for the factors walkability and quality of the environment, with a strong relationship for walkability |
| Vardoulakis | 2018 | Local action on outdoor air pollution to improve public health | Guidelines |  |  | Policy measures on transportation | Air pollution | The provided guidelines on transportation (infrastructure) can bring multiple public health benefits, including road accident prevention, carbon emissions reduction, improved physical activity levels, enhanced neighbourhood appearance and community cohesion, in addition to air pollution reductions. |
| Verhoeven | 2016 | Promoting Active Transport in Older Adolescents Before They Obtain Their Driving Licence: A Matched Control Intervention Study | Randomised control study | Youth (adolescents, teenagers) |  | Promotion of active travel | Awareness of the benefits of active travel | Intervention was not effective in changing psychosocial factors related to active transport. |
| Vienneau | 2015 | Years of life lost and morbidity cases attributable to transportation noise and air pollution: A comparative health risk assessment for Switzerland in 2010 | Cross sectional study |  |  | Pollution and noise exposure | Years of life lost | Estimations : in 2010 in Switzerland transportation caused 6000 years of life lost (YLL) from noise and 14 000 from pollution exposure. |
| Vuillemin | 2011 | Worksite physical activity interventions and obesity: a review of European studies (the HOPE project) | Miscelaneous type of review | Adults | 18y + | Active travel | fitness, obesity related-outcomes | Moderate evidence of effectiveness was found for physical activity outcomes with active commuting interventions. No or inconclusive evidence for obesity-related outcomes for all intervention categories. |
| Wang | 2010 | Occupational, commuting, and leisure-time physical activity in relation to heart failure among finnish men and women | Cross sectional study | Adults |  | Physical activity | Heart failure | Moderate or high levels of occupational or leisure-time physical activity have a negative association with the risk of heart failure among men and women |
| Wanner | 2012 | Active transport, physical activity, and body weight in adults: a systematic review | Systematic review | Adults |  | Active travel | Physical activity, BMI | Limited evidence that active transport is associated with more physical activity as well as lower body weight in adults. |
| Wennberg |  | Reduced risk of myocardial infarction related to active commuting: inflammatory and haemostatic effects are potential major mediating mechanisms | Cross sectional study | Adults; Older people |  | Active travel | Risk of myocardial infarction infarction | Car commuting was significantly associated with myocardial infarction risk |
| Wilkie | 2018 | Correlates of intensity-specific physical activity in children aged 9-11 years: a multilevel analysis of UK data from the International Study of Childhood Obesity, Lifestyle and the Environment | Cross sectional study | Children | 9-11y | Active travel | Physical activity | active transport was associated with meeting the physical activity guidelines |
| Woodcock | 2018 | Development of the Impacts of Cycling Tool (ICT): A modelling study and web tool for evaluating health and environmental impacts of cycling uptake | Modelling study | Mixed |  | Cycling | Health impacts | Tool to locally investigate the health impacts of a modal change from car-riding to cycling |
| Yang | 2021 | The effects of traveling in different transport modes on galvanic skin response (GSR) as a measure of stress: An observational study | Cross sectional study | Adults | 18-65y | Travel mode | Stress | Cycling and walking both reduce galvanic skin response (proxy measure for stress) while motorized modes of transportation increases it. |
